# Supplementary material for: Lasting antibody and T cell responses to SARS-CoV-2 in COVID-19 patients three months after infection
Source: Nat Commun. 2021 Feb 9;12:897. doi: 10.1038/s41467-021-21155-x (PMC7873066; doi:10.1038/s41467-021-21155-x)
Supplement: Supplementary file 1 — Supplementary Information [file 41467_2021_21155_MOESM1_ESM.pdf]

Supplementary information

Lasting Antibody and T cell Responses to SARS-CoV-2 in COVID-19 Patients

Three Months After Infection

Jiang *et al*.

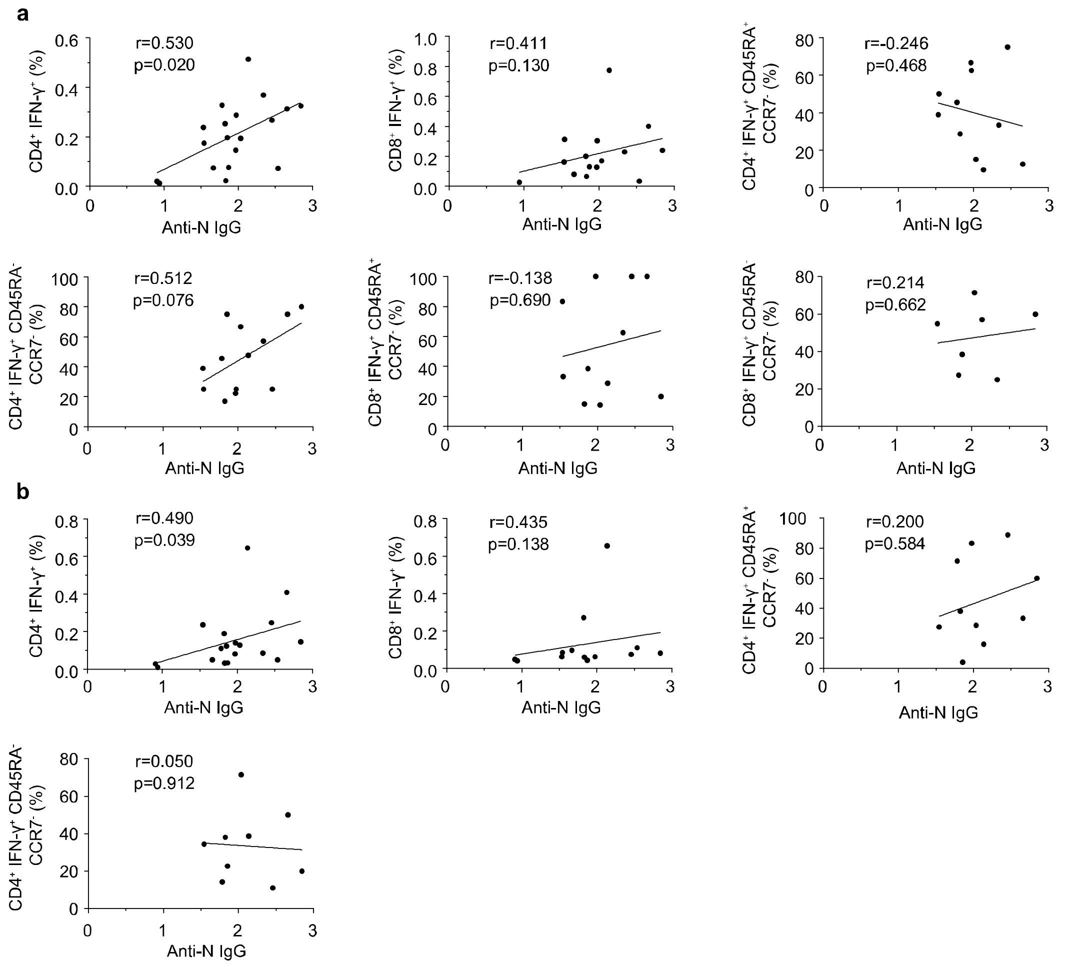

Supplementary Fig. 1. Correlations between SARS-CoV-2-specific CD4 and CD8 T cell response and IgG response to nucleoprotein. a, b Correlation between SARS-CoV-2 nucleoprotein (**a**) and spike (**b**) specific $CD4^+$ and $CD8^+$ T cells (%) and anti-N IgG. Statistical comparisons were performed using two-sided nonparametric Spearman correlation. p value and spearman's rho are presented.

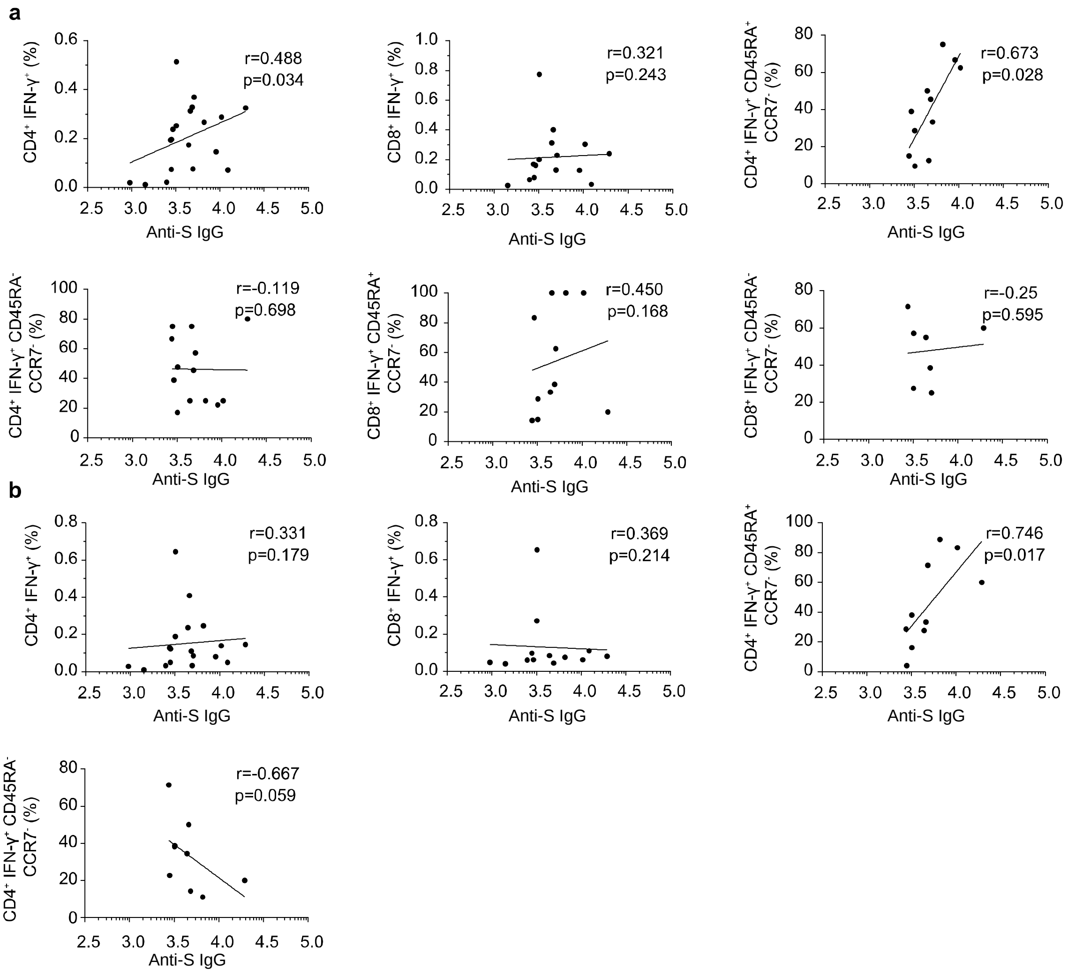

Supplementary Fig. 2. Correlations between SARS-CoV-2-specific CD4 and CD8 T cell response and IgG response to spike. a, b Correlation between SARS-CoV-2 nucleoprotein (N) (**a**) and spike (S) (**b**) specific $CD4^+$ and $CD8^+$ T cells (%) and anti-S IgG. Statistical comparisons were performed using two-sided nonparametric Spearman correlation. p value and spearman's rho are presented.

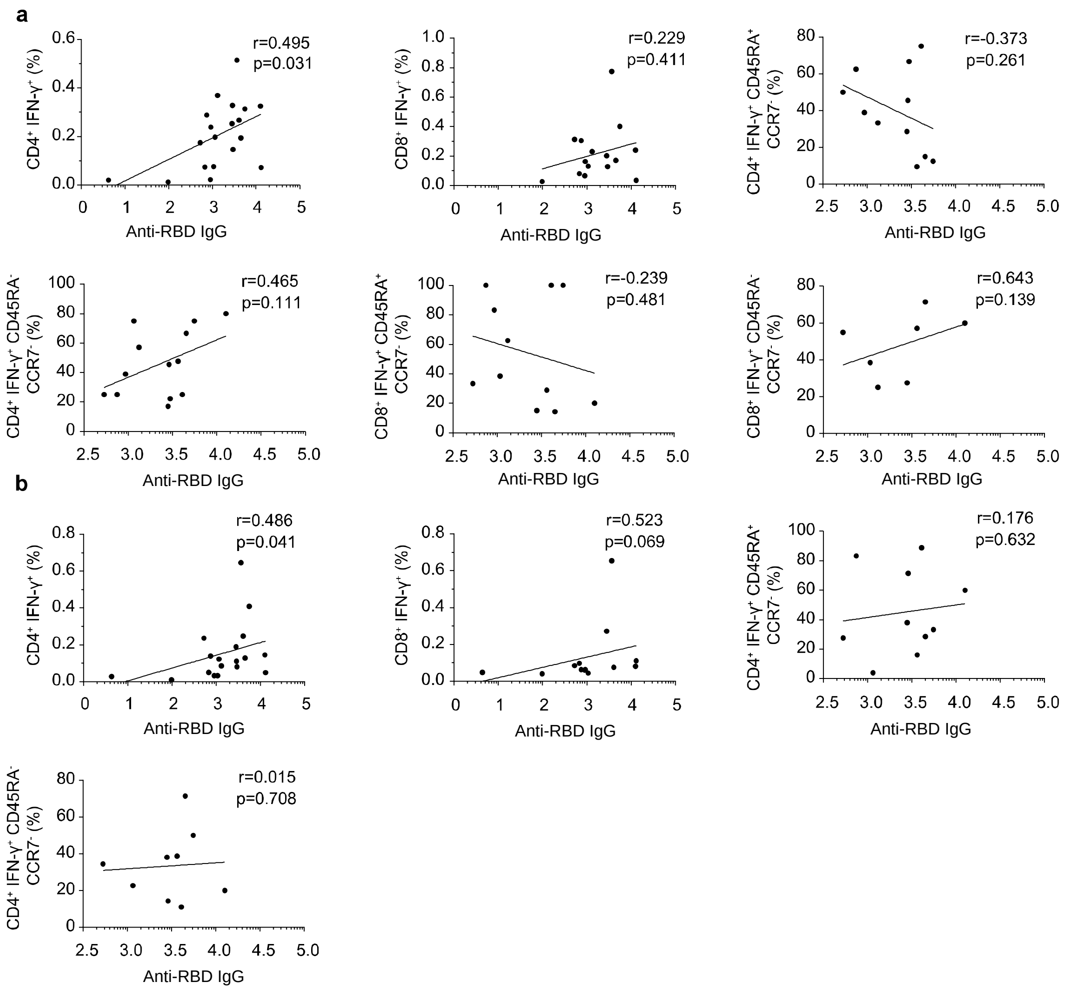

Supplementary Fig. 3. Correlations between SARS-CoV-2-specific CD4 and CD8 T cell response and IgG response to the receptor-binding domain of spike. a, b Correlation between SARS-CoV-2 nucleoprotein (**a**) and spike (**b**) specific $CD4^+$ and $CD8^+$ T cells (%) and anti-receptor binding domain (RBD) IgG. Statistical comparisons were performed using two-sided nonparametric Spearman correlation. p value and spearman's rho are presented.

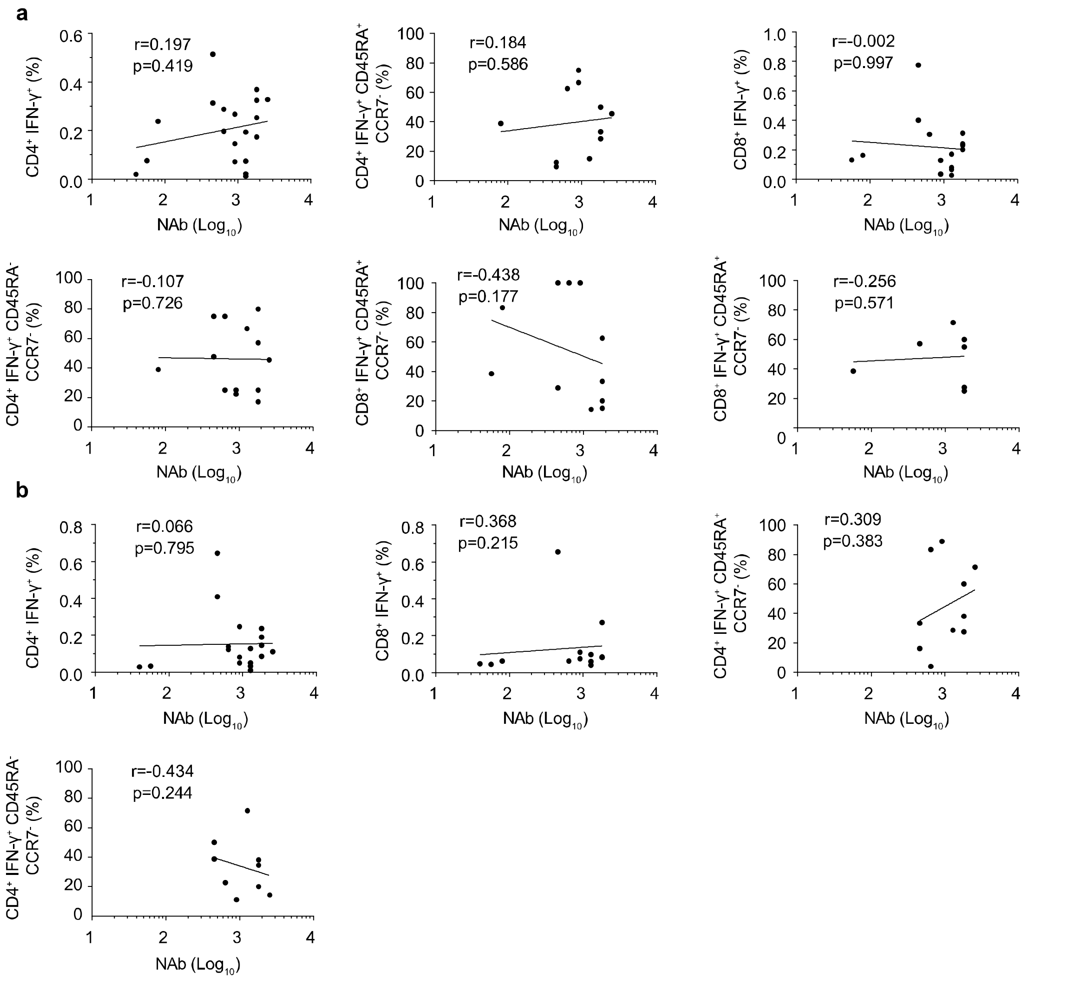

Supplementary Fig. 4. Correlations between SARS-CoV-2-specific CD4 and CD8 T cell response and neutralizing antibodies (NAb) a, b Correlation between SARS-CoV-2 nucleoprotein (**a**) and spike (**b**) specific CD4^{+} and CD8^{+} T cells (%) and NAb titer. Statistical comparisons were performed using Spearman correlation. p value and spearman's rho are presented.

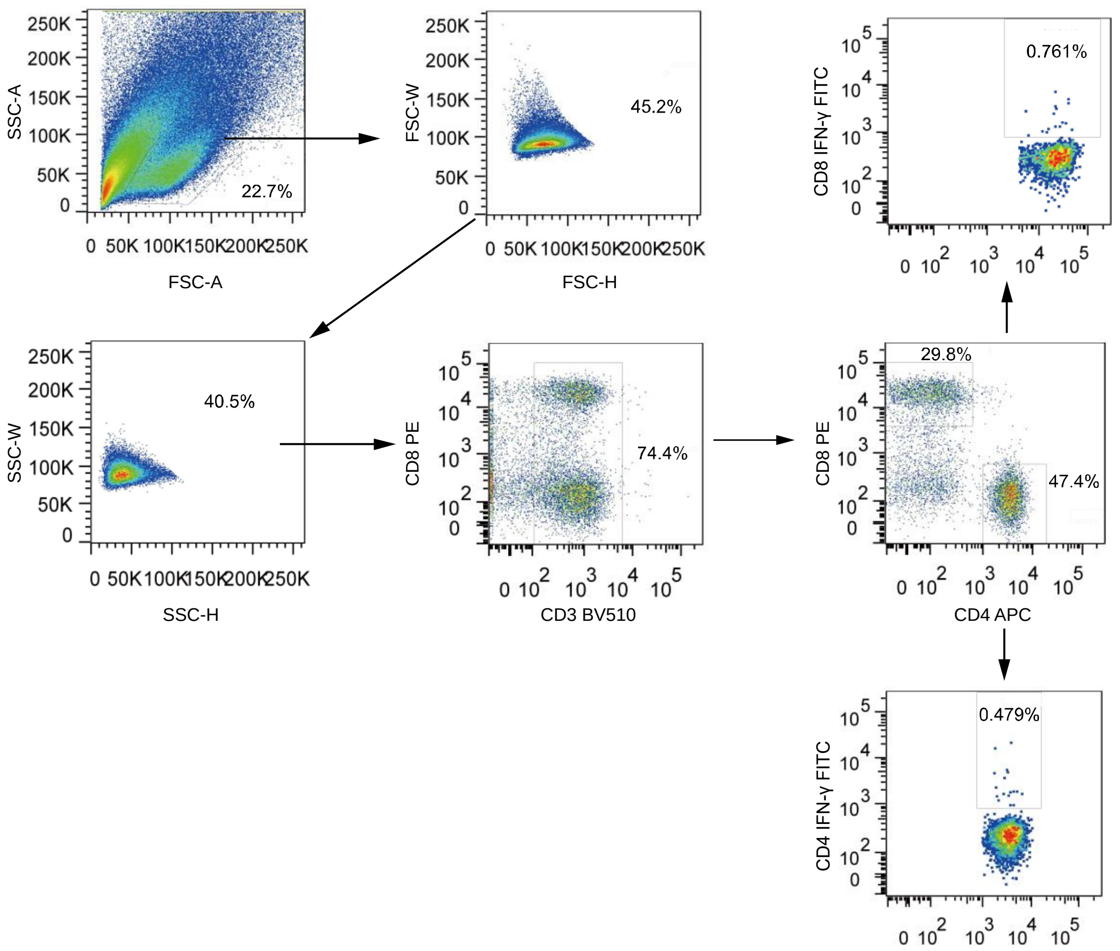

Supplementary Fig. 5. The gating strategy of T cells subsets. The gating strategy is shown for one representative patient. T-cell subsets were identified via the following gating strategy: lymphocytes (FSC/SSC), doublets (FSC-H/FSC-W), singlets (FSC-A/FSC-H), LIVE $CD3^+$ cells were selected and divided in $CD3^+CD4^+$ and $CD3^+CD8^+$. Plots for IFN-γ are shown for $CD4^+$ and $CD8^+$ T cells from one representative patient. Within the CD4 and CD8 subsets, memory subsets were gated as $CD45RA^+CCR7^+$ (naïve), $CD45RA^-CCR7^+$ (central memory), $CD45RA^-CCR7^-$ (effector memory) or $CD45RA^+CCR7^-$ (late effector memory), and the gating strategy is shown in Fig 4.
