# Supplementary material for: Lasting antibody and T cell responses to SARS-CoV-2 in COVID-19 patients three months after infection
Source: Nat Commun. 2021 Feb 9;12:897. doi: 10.1038/s41467-021-21155-x (PMC7873066; doi:10.1038/s41467-021-21155-x)
Supplement: Supplementary file 3 — Reporting Summary [file 41467_2021_21155_MOESM3_ESM.pdf]

nature research

Corresponding author(s): Mai-juan Ma

Last updated by author(s): Dec 30, 2020

Reporting Summary

Nature Research wishes to improve the reproducibility of the work that we publish. This form provides structure for consistency and transparency in reporting. For further information on Nature Research policies, see our Editorial Policies and the Editorial Policy Checklist.

Statistics

For all statistical analyses, confirm that the following items are present in the figure legend, table legend, main text, or Methods section.

n/a	Confirmed	
☐	☒	The exact sample size (*n*) for each experimental group/condition, given as a discrete number and unit of measurement
☐	☒	A statement on whether measurements were taken from distinct samples or whether the same sample was measured repeatedly
☐	☒	The statistical test(s) used AND whether they are one- or two-sided<br>*Only common tests should be described solely by name; describe more complex techniques in the Methods section.*
☒	☐	A description of all covariates tested
☒	☐	A description of any assumptions or corrections, such as tests of normality and adjustment for multiple comparisons
☐	☒	A full description of the statistical parameters including central tendency (e.g. means) or other basic estimates (e.g. regression coefficient) AND variation (e.g. standard deviation) or associated estimates of uncertainty (e.g. confidence intervals)
☐	☒	For null hypothesis testing, the test statistic (e.g. *F*, *t*, *r*) with confidence intervals, effect sizes, degrees of freedom and *P* value noted<br>*Give P values as exact values whenever suitable.*
☒	☐	For Bayesian analysis, information on the choice of priors and Markov chain Monte Carlo settings
☒	☐	For hierarchical and complex designs, identification of the appropriate level for tests and full reporting of outcomes
☐	☒	Estimates of effect sizes (e.g. Cohen's *d*, Pearson's *r*), indicating how they were calculated

Our web collection on statistics for biologists contains articles on many of the points above.

Software and code

Policy information about availability of computer code

Data collection	No software was used for data collection.
Data analysis	Data were analysed in Prism 7.0 and FlowJo v10.

For manuscripts utilizing custom algorithms or software that are central to the research but not yet described in published literature, software must be made available to editors and reviewers. We strongly encourage code deposition in a community repository (e.g. GitHub). See the Nature Research guidelines for submitting code & software for further information.

Data

Policy information about availability of data

All manuscripts must include a data availability statement. This statement should provide the following information, where applicable:

- Accession codes, unique identifiers, or web links for publicly available datasets
- A list of figures that have associated raw data
- A description of any restrictions on data availability

The data that support the findings of this study are available within the paper and its supplementary information files. Source data for figures are provided with this paper.

Field-specific reporting

Life sciences study design

All studies must disclose on these points even when the disclosure is negative.

Sample size	The aim of this this study was to characterize the dynamic of SARS-CoV-2 specific antibody and T cell response three-four months after infection. We enrolled 25 COVID-19 patients. Of these 25 patients, 16 were newly diagnosed, and their blood samples were collected at multiple timepoints up to 3-4 months after infection. Additional 9 were recovered patients 3-4 months after infection was also enrolled and their blood samples were collected at this one timepoint. No prior sample size calculation was performed since sample size was determined by the number of patients identified in the the local at the early time point and 3-4 months after infection.
Data exclusions	No data was excluded from the analyses.
Replication	The authors guarantee the findings are reliably reproducible. All the laboratory tests are runned in duplicate.
Randomization	Randomization was no applicable in this study since all patients who were willing to participante was enrolled non-selectively during the first outbreak of SARS-CoV-2 in Linyi City, Shandong Province, China.
Blinding	ELISA, neutralization assay, and flow cytometry were performed independently by researchers blind to samples information. Blinding was not performed during flow cytometry gating analysis because gates were applied in an unbiased manner across all samples.

Reporting for specific materials, systems and methods

We require information from authors about some types of materials, experimental systems and methods used in many studies. Here, indicate whether each material, system or method listed is relevant to your study. If you are not sure if a list item applies to your research, read the appropriate section before selecting a response.

Materials & experimental systems

n/a	Involved in the study
☐	☒ Antibodies
☐	☒ Eukaryotic cell lines
☒	☐ Palaeontology and archaeology
☒	☐ Animals and other organisms
☐	☒ Human research participants
☒	☐ Clinical data
☒	☐ Dual use research of concern

Methods

n/a	Involved in the study
☒	☐ ChIP-seq
☐	☒ Flow cytometry
☒	☐ MRI-based neuroimaging

Antibodies

Antibodies used	For flow cytometry<br>Biolegend antibodies: BV510-labeled anti-CD3 (300448, UCHT1), APC-labeled anti-CD4 (357408, A161A1), PE-labeled anti-CD8a (300908, HIT8a), PE-Cy7-labeled anti-CD45RA (304126, HI100), BV421-labeled anti-CCR7 (353208, G043H7), PerCP-Cy5.5-labeled anti-TNF-α (502926, MAb11), and BV421-labeled anti-GzmB (396414, QA18A28).<br>BD Biosciences antibodies: FITC-labeled anti-IFN-γ (11-7319-82, 4S.B3)<br><br>For spike and RBD ELISA<br>HRP-conjugated goat anti-human IgG (W4031, 1:5000, Promega)
Validation	Antibody validations were performed by the suppliers per quality assurance literature provided by each supplier. All respective validation data are available on the manufacture's website.

Eukaryotic cell lines

Policy information about cell lines

Cell line source(s)	Vero E6 cells was obtained from ATCC.
Authentication	Authentication was performed regularly based on morphology and gene/protein expression (in case of genetic alterations)
Mycoplasma contamination	Vero E6 cells tested mycoplasma negative.
Commonly misidentified lines<br>(See ICLAC register)	No commonly misidentified cell lines were used in the study.

Human research participants

Policy information about studies involving human research participants

Population characteristics	Twenty-five laboratory-confirmed SARS-CoV-2 patients were enrolled. Of the 25 enrolled patients, 3 were severe patients, 18 were moderate patients, and 4 were asymptomatic (Table 1). The median age was 40 years (interquartile range [IQR], 33–53), and 13 (52%) were male. Fever and cough were the most common symptoms reported. Seventy-two percent of patients experienced moderate illness. Fifty-two percent of these individuals had known underlying medical illnesses.
Recruitment	All the available COVID-19 patients at the early time point and 3-4 months after infection who were will to participate in this study were recureited in this study. No-selection bias existed to the best of our knowledge.
Ethics oversight	Written informed consent for participation in this study was obtained from all adult participants or guardians on behalf of the children enrolled in this study. The study was conducted following the Declaration of Helsinki, and the Institutional Review Board of the Academy of Military Medical Sciences approved the study protocol (IRB number: AF/SC-08/02.46)

Note that full information on the approval of the study protocol must also be provided in the manuscript.

Flow Cytometry

Plots

Confirm that:

- [x] The axis labels state the marker and fluorochrome used (e.g. CD4-FITC).
- [x] The axis scales are clearly visible. Include numbers along axes only for bottom left plot of group (a 'group' is an analysis of identical markers).
- [x] All plots are contour plots with outliers or pseudocolor plots.
- [x] A numerical value for number of cells or percentage (with statistics) is provided.

Methodology

Sample preparation	PBMC were isolated from whole blood and cryopreserved. Samples were thawed in batches and either stimulated (T cell data).
Instrument	Samples were acquired on a BD FACSCalibur.
Software	FlowJo version 10
Cell population abundance	N/A. No sorting was performed.
Gating strategy	T-cell subsets were identified via the following gating strategy: lymphocytes (FSC/SSC), doublets (FSC-H/FSC-W), singlets (FSC-A/FSC-H), LIVE CD3+ cells were selected and divided in CD3+CD4+ and CD3+CD8+. Plots for IFN-γ are shown for CD4+ and CD8+ T cells from one representative patient. Within the CD4 and CD8 subsets, memory subsets were gated as CD45RA +CCR7+ (naive), CD45RA-CCR7+ (central memory), CD45RA-CCR7- (effector memory) or CD45RA+CCR7- (late effector memory). Th gating strategy is also shown in supplementary Fig 5 and Fig 4.

- [x] Tick this box to confirm that a figure exemplifying the gating strategy is provided in the Supplementary Information.
